# Ambiguous Compliments and Encouragement: Young Fathers’ Experiences of Care, Responsibility and Youth

**DOI:** 10.1007/s10560-026-01118-1

**Published:** 2026-05-29

**Authors:** Marijke Sniekers, Lieke Tillmann, Milotte Hamer

**Affiliations:** Zuyd University of Applied Sciences, Heerlen, Netherlands

**Keywords:** Young fathers, Fatherhood, Gender norms, Masculinity, Age, Youth, Care Work, Participatory Action Research, Social Work

## Abstract

Even though fathers matter in the lives of their children, their partners and in society, engaging fathers in social work services has remained hard to follow through. Our participatory action research project in a youth care network in the Netherlands aimed at strengthening the inclusion of young fathers in youth care practice. This article explores how young fathers experience their fatherhood in relation to gender and age norms. Their narratives reveal that age, gender and ethnicity intersect to shape norms of youthfulness from which young fathers deviate by positioning themselves as mature. The young fathers describe their care work as childcare and as relational work, while aligning these care practices with traditional norms of masculinity such as providership and responsibility. Although caring fatherhood is increasingly recognised, they experience ambivalent reactions on community and institutional levels. They receive ambiguous compliments and encouragement that evoke feelings of stigma. Their fatherhood brings positive changes, yet structural barriers in social work systems persist. They often feel unrecognised by social work professionals. The project, however, contributed to professional development through an enhanced father-inclusive support method and training programme. By closely looking into the fathers’ perspectives, this article calls for a shift in social work practice and policy. Findings underline that meaningful engagement with young fathers requires addressing institutional norms, ensuring equal recognition of fathers and mothers, and adopting gender- and age-sensitive approaches that acknowledge the diverse realities of young fathers.

## Introduction

Young fatherhood is widely studied as a complex transition of adolescents navigating emotional, financial and relational challenges (Neale, 2016; Neale & Tarrant, 2024; SmithBattle et al., 2019; Uengwongsapat et al., 2018). It is understood through both a ‘social problems’ and a ‘social engagement’ framework (Neale & Tarrant, 2024). On the one hand, young fathers are more often vulnerable and underprivileged than their childless peers, are more frequently raised in deprived circumstances and more often experience mental issues (Deslauriers & Kiselica, 2024). Although young fathers themselves do not regard their age as an obstacle to assuming a paternal role, judgement of others affects them (Deslauriers & Kiselica, 2024; Rice et al., 2019). Young fathers are stigmatised for not having the abilities to care for their children, for depending on welfare benefits, being irresponsible and having had unprotected, casual sex at a too early age (Beggs Weber, 2020). They experience double stigma, when norms on gender and age intersect. Being young and being men caring for their children marginalises them. On the other hand, studies portray engaged and involved young fatherhood (Andreasson et al., 2022; Neale & Tarrant, 2024; Reinicke, 2021). Fatherhood can offer a sense of purpose and responsibility, motivating young men to improve their lives to be good fathers (Deslauriers & Kiselica, 2024; Neale & Tarrant, 2024; Reinicke, 2021). Young fathers take up paternal responsibilities and care (Deslauriers & Kiselica, 2024). They are more responsible, actively involved in their children’s lives and protect their family (Beggs Weber, 2020; Neale, 2016). These contrasting frameworks indicate that young fatherhood may be experienced positively from an individual and family perspective, while being framed as problematic from societal and institutional perspectives. This contrast calls for further exploration of norms on young fatherhood across individual, community and institutional perspectives. It is well-known that fathers matter in the lives of their children, their partners and in society. Children, mothers, fathers themselves and society benefit socially, cognitively, economically, and emotionally from fatherly involvement (Distelbrink et al., 2023; Grau Grau et al., 2022; Keizer et al., 2020). Even though the care work of men in heterosexual relationships has increased and has become more diverse, compared to that of women, men still provide less care work (Adler, 2023; Eerola et al., 2025; Van der Gaag et al., 2023). A ‘male provider/female carer’ model is often still seen as the ‘ideal’ family structure in many societies (Harthoorn, 2019; Martínez-Pastor et al., 2024; Van der Gaag et al., 2023). Moreover, youngsters reflect these traditional ideals and norms in their narratives on care and responsibility (Bonner-Thompson & Nayak, 2022; Scholz et al., 2025). Young fathers typically identify a ‘good father’ as a financial provider, or an emotionally present caregiver (Andreasson et al. 2022; Tarrant et al. 2025a; Paschal et al. 2011). However, fulfilling this ideal can be constrained by limited educational attainment, financial insecurity, unemployment or a lack of positive role models and support (Neale and Davies 2015; Reinicke 2021; SmithBattle et al. 2019; Tarrant et al. 2025a). The need for fathers’ involvement has been widely recognised in social work and health care, but engaging fathers in support and care services has remained hard to follow through (Grau Grau et al., 2022; Pfitzner et al., 2020; Webb et al., 2023). Young fathers are generally labelled as hard to reach, which points the blame towards themselves, while in reality services are poorly adapted to their lives and realities (Baum 2017; Davies 2016; Mniszak et al. 2020; Neale and Davies 2015; Tarrant et al. 2025a). Although policies aim to be inclusive, they often target mothers, and services and programmes that target parents are usually attended by mothers only (Daly 2013; Grau Grau et al. 2022; Pfitzner et al. 2020; Webb et al. 2023; Tarrant et al. 2025b). Fathers themselves often consider parental services and programmes to be for mothers only and do not find their needs met (Daly, 2013; Edwards & Gillies, 2011; Mniszak et al., 2020). Fathers are poorly acknowledged and excluded by organisations and professionals who (unintentionally) uphold dominant gender norms of women as carers and fathers as providers (Webb et al., 2023). Professionals, mostly women, often struggle to connect with fathers, partly due to mutual discomfort, stereotyped gender roles, and the perception that ‘strong men’ do not need support (Baum, 2017). Care professionals see fathers less often than mothers, pay less attention to fathers and speak of fathers in more general, remote and stereotypical terms (Veltkamp & Brown, 2017). Support for *young* fathers is even less available (Mniszak et al., 2020). They feel even more out of place and unwelcome in social work or health care services (Pfitzner et al. 2020; Tarrant et al. 2025b; Van de Beek et al. 2025b). Young fathers’ experiences thus need to be uncovered, in order for social work services and professionals to develop father-inclusive professional practices.

This study adds to the growing body of international literature on young fatherhood, by focusing on young fathers’ experiences in the Netherlands. Research on (young) fatherhood in the Netherlands is limited. The Netherlands has relatively many men in care, compared to other European countries (Martínez-Pastor et al., 2024). Yet unlike other countries in Europe, a one-and-a-half earner model is common, with fathers working full-time and mothers switching to part-time work when the couple has children (Amman & Vermuë, 2024; Van den Brakel & Bruggink, 2024). This is gradually changing, but, despite government efforts in 2020 to prolongate paternity leave, many fathers do not fully use it, and mothers still take up most caring and household responsibilities (Amman & Vermuë, 2024; Harthoorn, 2019; Van den Brakel & Bruggink, 2024).

Youngsters in the Netherlands are considered ‘young’ in socio-economic and education policy until they are 28; they must attend school, have attained basic secondary level education or participate in a welfare-to-work programme (Levels et al., 2020; Nederland et al., 2016). Social work organisations in the Netherlands often support youngsters under the age of 28 who have not met these objectives. Especially when they have children, they are eligible for youth care or fall under the responsibility of child protection services. Nevertheless, social work organisations often overlook (young) fathers (Borman, 2022; Sniekers et al., 2023).

To include the perspectives of young fathers more and engage with them, youth care professionals and researchers in the south of the Netherlands have set up a participatory action research project. This article derives from this project and aims to shed light on young fathers’ experiences of fatherhood in a mother-centred culture on individual, institutional and normative levels. The research question is: In which ways do young fathers in the south of the Netherlands experience their young fatherhood in relation to gender and age norms? By foregrounding young fathers’ perspectives, it seeks to expose the interactions of gender and age at multiple societal levels. Building on these insights, the participatory action undertaken has led to strengthening the support method used by youth care workers, improving their engagement with and support of young fathers. This process has enhanced social workers’ professional development and fostered more gender-sensitive and father-inclusive attitudes and practices.

The next section sets out the theoretical underpinnings of the study, followed by the methods section explaining the participatory action research and methods relevant for this article. Next, the results present intersecting norms of gender and age in young fathers’ experiences and practices of their fatherhood from individual, community and institutional perspectives. A conclusion and discussion section ends the article with a call for father-inclusive social work practice and policy and provides relevant suggestions.

## Theoretical Framework

Whereas femininity is intertwined with motherhood, masculinity is not necessarily related to fatherhood. Women are expected to become mothers and studies have focused on mothering skills and ‘good motherhood’ (Arendell, 2000; Phoenix et al., 1991; Smyth, 2012). Fathers have long been considered as main providers, less capable caregivers and as the ‘second parent’ (Gallais, 2023). Scholars increasingly have shown engaged, involved and caring fatherhood, which goes against the masculinity ideal of strong, unemotional and authorative men (Andreasson et al., 2023; Grau Grau et al., 2022; Hunter et al., 2017; Wojnicka & De Boise, 2025). This has built an identity of fathers as authoritarian, disinterested, absent and emotionally distant (Hunter et al., 2017; Wojnicka & De Boise, 2025). This masculinity ideal, however, is not fixed, but constituted through practices enacted across diverse social actions, gender relations and settings (Connell & Messerschmidt, 2005). Scholarship on caring masculinities states that more men are moving away from male dominance, privilege and power, toward care work, emotional engagement, relationality and interdependence in their masculine identity and practices (Eerola et al., 2025; Elliott, 2016; Hanlon, 2024; Roberts & Prattes, 2024; Wojnicka & De Boise, 2025). This change towards care inclusive masculinity can either be seen as broadening the masculinity ideal or as unsettling it (Nayak, 2023; Wojnicka & De Boise, 2025). More attention is needed for how young fathers navigate this normative spectrum, exploring whether they contest or comply with prevailing norms.

Young fatherhood is often perceived as an accident, and young fathers are generally seen as absent (Deslauriers & Kiselica, 2024). However, decision-making processes on pregnancies are layered and complex, and young men’s intentions of becoming a father can vary from an explicit, ambivalent or latent desire to absence of a desire to be a father (Beumer et al., 2023; Dalmijn et al., 2024; Deslauriers & Kiselica, 2024; Van de Beek et al. 2025a). Although the ultimate decision to continue a pregnancy into term or not mostly lies with mothers, fathers play a crucial role (Dalmijn et al., 2024; Deslauriers & Kiselica, 2024). Their support to the mother, a stable and safe relationship and their positive attitude about the pregnancy strongly contribute to carrying a pregnancy into term (Dalmijn et al., 2024). Fear and shame or stigma about abortion, on the other hand, negatively impact the decision (Dalmijn et al., 2024).

Unlike older, privileged fathers who rarely report stigma (Ammann & Vermuë, 2024), young fathers often feel judged and unsupported (Rice et al. 2019; SmithBattle et al. 2019; Tarrant et al. 2025a). Moreover, stigma that young fathers experience differs from that of young mothers, due to the different gender and parenting norms for women and men (Beggs Weber, 2020; Neale & Tarrant, 2024). Although in many societies, masculinity is the norm and considered as a neutral position (Wekker, 2002), this is not the case for the context of parenthood, where motherhood is the perceived neutral and default position. Intersectional studies on fatherhood show that a focus on white, middle-class fathers can lead to negative stereotyping of men who do not fit this ideal father image (Ammann & Vermuë, 2024; Distelbrink et al., 2023; Strier & Nadav-Vaisvidosky, 2021). To challenge such assumptions of neutrality and explore norms and stigma, an intersectional approach may be useful.

Intersectionality is widely used as a theoretical and analytical framework across disciplines (Crenshaw, 1991; Davis, 2008; Hill-Collins, 2015; Smiet, 2017). It examines how socially constructed identity categories, such as gender, age, ethnicity or class, intersect and are enacted through everyday practices and institutional norms, producing complex experiences of privilege, inequality, and exclusion (Crenshaw, 1991; Lutz et al., 2011; West & Zimmerman, 1987; West & Fenstermaker, 1995). Lutz et al. (2011) distinguish three analytical levels at which identity categories manifest and acquire meaning. The personal level refers to how young fathers experience and construct their identities, and which identity aspects and characteristics are assigned to young fathers by others. The institutional level relates to how they are seen by professionals and are positioned within institutions and organisational structures, and how organisations are structured differently. The symbolic level concerns how gender and age norms are differently valued. These values can be positive, but they can also be negative, leading to stigma. The dimensions are not mutually exclusive and the symbolic level interacts with the personal and institutional. Drawing from this approach enables us to examine the multiple levels at which norms on youth, fatherhood and masculinity shape young fathers’ identities.

Although the word ‘parent’ is often perceived as a gender-neutral term, research shows that fathers feel less addressed by this term than what is generally intended in parenting policy and parenting literature (Borman, 2022; Edwards & Gillies, 2011). In professional parenting theory, ‘parenting’ is used as a neutral or inclusive term. It is explained as unconditional and timeless, with parenthood being an identity, instead of a role or task (Remmerswaal & De Gouw, 2024; Van der Pas, 2016). The parenting theory of Van der Pas (2016; Remmerswaal & De Gouw, 2024) is based on three premises for professionals to keep in mind when working with parents: (1) every parent has a sense of responsibility and wants to do what is right for their child; (2) parenting makes vulnerable, because no parent is perfect; and (3) parents are ultimately responsible, while professionals are passers-by to support in times of need. These premises may help to reduce professional and institutional stigma around young fatherhood, by normalising paternal responsibilities and absolving fathers of mistakes and feelings of inadequacy that are usually framed as individual faults.

However, this parenting theory speaks of parenting rather than fathering or mothering (Remmerswaal & De Gouw, 2024; Van der Pas, 2016). Especially when examining aspects of a resilience system of parents that provide protection in challenging circumstances, such as a clear division of tasks or a supportive community (Van der Pas, 2016), parenting cannot be regarded as a gender-neutral practice, since paternal and maternal practices are closely tied to notions and practices of masculinity and femininity (Adler, 2023). Using ‘parenting’ instead of ‘mothering’ and ‘fathering’ does not account for the social embeddedness, the relational division of labour and the lived, meaningful relationship between the mother or father and the child (Daly, 2013; Edwards & Gillies, 2011). We acknowledge that the term ‘parent’ exceeds the dominant binary of mother/father with, for example, co-parents, step-parents, foster-parents, non-birthing parents, adoptive parents and non-parental caregivers. Even though diversities in family structures go beyond the traditional image of a heterosexual nuclear family, this does not detract from the need to explicate practices and experiences of fathers and fatherhood.

## Methods

### Research Context

The research is set in the south of the Netherlands, concentrated in Parkstad region. This area faces socio-economic challenges such as poverty, lower educational, literacy and numeracy levels, high unemployment, higher welfare dependency and higher youth care provision than on average in the Netherlands (Hooijen et al., 2022). The high percentage of youth in care and protection services is influenced by interacting factors such as parental mental problems, domestic violence and fathers who are not involved (Geijtenbeek et al., 2019; Hooijen et al., 2022). The regional government has committed to break the intergenerational cycle of poverty through long-term programmes and projects. Several youth care organisations in the region have adopted a systemic and strengths-based approach (Rapp & Goscha, 2012), grounded in positive psychology (Seligman & Csikszentmihaly, 2000). These youth care professionals that aimed to support young parents, though, mainly supported young mothers. They considered young fathers “hard to reach” and noted they were “not in the picture” and “not involved in the mothers’ or children’s lives” (Sniekers et al., 2023). The professionals aspired to support young fathers too. Therefore, these youth care providers and a university of applied sciences set up a participatory action research (PAR) project with young fathers and mothers. It aimed to strengthen the parenthood and pedagogical climate in young families and increase social participation of young parents, by broadening the professional support method for young mothers to also include and engage young fathers.

### Participatory Action Research

The PAR project involved broad participation of youth care professionals, researchers, young fathers and mothers and social work students, who took actions during the research process to change the existing situation (Van Lieshout et al., 2017). Furthermore, it enabled partners to be co-creative participants who carried out various roles and tasks, following the common PAR phases of collaborative planning, action, reflection and evaluation, over a period of four years (Van Lieshout et al., 2017).

The existing learning community of 22 youth care professionals (twenty women, two men) was expanded with two researchers (women) and one process facilitator (man). This stimulated mutual learning of professional practice and conducting research (Cornish et al., 2023). The learning community met monthly for care deliberations, peer learning, thematic discussions as ways of reflexivity. Participants developed a joint mission statement and vision, held dialogue sessions with young fathers and mothers, and provided feedback on products. This helped building relationships, establishing working practices, gathering data, planning and taking joint action (Cornish et al., 2023). They also developed workshops for yearly conferences to share the proceedings with other professionals and policymakers.

Additionally, a working group of five youth care professionals and two researchers (all women) was formed within the learning community. These participants co-developed and conducted data gathering and analysis, strengthened the existing support method, and co-developed and delivered the related training. Each year, several different bachelor Social Work students (women and men) participated as co-researchers. Together with young fathers and mothers, they also developed tools for the support method and training. This fostered the establishment of working practices and a collaborative research and practice development (Cornish et al., 2023). It also helped to decentralise control and share power (Cornish et al., 2023).

A new advisory group was established with two young mothers and one young father, one youth care professional (woman), one researcher (woman) and five professionals in health care and social policy (four women, one man). They co-developed the research tools and informed consent forms with the working group, and provided feedback on products, support method and training. This facilitated common understandings of issues, build relationships between participants and developed working practices (Cornish et al., 2023). The advisory group also cultivated a critical community to hold the working group accountable (Cornish et al., 2023).

### Dialogue Sessions

The presented findings are derived from dialogue sessions between professionals and young fathers and mothers in the first phase of the PAR. Dialogue, reciprocity, and ownership are central to youth-focused research and practice (Mann et al., 2014; Wylie, 2014). Young parents’ views and experiences were considered equally important as those of the professionals, and the term ‘dialogue session’ emphasised an open, co-reflective exchange. Calling it an interview would also be a barrier for fathers and mothers, suggested a mother and father in the advisory group. Professionals used a topic guide, that was co-developed by the advisory group. The sessions intended to identify experiences of young parents and professionals as foundational experiential knowledge to redevelop the support method and to design a training programme for professionals in a next PAR phase.

Dialogues sessions offer advantages and disadvantages over individual interviews (Bjørnholt & Farstad, 2014; Szulc & King, 2022; Zarhin, 2018). An advantage was that they encouraged richer detail without the professional prompting, making it more of a three-way conversation (Szulc & King, 2022; Zarhin, 2018). They added depth as partners responded or provided new insights to each other, and allowed partners greater control over their shared narrative (Bjørnholt & Farstad, 2014; Szulc & King, 2022; Zarhin, 2018). However, disadvantages were social desirability, silencing or overshadowing of one partner, or conflict between partners (Bjørnholt & Farstad, 2014; Szulc & King, 2022; Zarhin, 2018). To mitigate these risks, researchers and professionals received training in facilitating open dialogue and equal sharing of perspectives, recognising interpersonal dynamics, and ensuring informed consent. Overall, the dialogue sessions were enriching. Fathers and mothers complemented and followed up on one each other’s accounts, no conflicts occurred, compliments were shared and reflection emerged. Professionals adopted a less directive role than in traditional interviews, but the parents were still reluctant to ask return questions. Practical considerations, such as meeting jointly at home when children were asleep, facilitated participation.

### Participants in Dialogue Sessions

Initially, young fathers and mothers supported by youth care professionals were invited to participate. Six couples were recruited this way. However, recruitment proved more difficult than expected. Professionals sometimes felt unable to ask fathers, due to concerns about disrupting support and trust, or because fathers were facing particularly challenging circumstances. To reach the target of ten fathers, the timeframe was extended and recruitment broadened beyond the PAR organisations to include another four young couples in the region, for example via the informal networks of (male) social work students and researchers. This approach was successful and increased the diversity of the sample.

The ten fathers that participated in the dialogue sessions were between 24 and 30 years old, and had their first child between the ages of 19 and 29. The mothers of their children were between 20 and 28 years old and had their first child between the ages of 18 and 25 years. One father was 29 at first childbirth and was considered too old initially. However, it was decided to include them as a couple, since they both received professional support from youth care professionals and his partner was younger. Four fathers had one child and six fathers had two children, one with two different mothers. One father was single, eight fathers had a partner and one was married. These nine fathers co-habitated with their partner. Seven fathers were born in the Netherlands and had a Dutch background. Three were born in the Caribbean and had a Surinamese or Antillean background. The focus here is on the fathers and results show their quotes.

Four youth care workers, one mental health worker and one maternal care specialist (women) participated in the dialogue sessions. Their work experience ranged from 12 to 32 years of working with children and (young) parents. They represented all PAR organisations, with two professionals from the same organisation.

### Data Collection and Analysis

Dialogue topics were fathers’ experiences of pride, parenting identity, tasks in parenting, experiences with care, norms on (young) fatherhood and possible stigma. All sessions were recorded and transcribed with informed consent, and approved by an external Medical Ethical Commission. Four researchers, including one professional trained as co-researcher, analysed the data using inductive open and selective coding in Atlas.ti (Evers, 2015). The analysis began with open coding by a junior and senior researcher, with one transcript independently coded by a Bachelor student and reviewed by the senior researcher. This was followed by selective coding in which open codes were categorised into key themes (Evers, 2015) such as responsibility, care, vulnerability, norms, stigma, support, network. The research team, including the professional co-researcher and student, discussed the codes to ensure consistency and trustworthiness. Interim results were discussed with the learning community and advisory group. Their feedback informed the final analysis, which laid the foundation for the following results, but was also incorporated into the development of the support method and training programme.

## Results

This section presents how young fathers in the Netherlands experience their fatherhood in relation to prevailing gender and age norms. Drawing on their perspectives, the findings highlight how gender and age based expectations and norms intersect across individual, institutional and symbolic levels.

### “Ahead of My Friends”: Age Norms On an Individual Level

The ways young fathers experience their fatherhood reflect various youth norms that become visible at an individual level. Fatherhood was not ‘a mistake’ for most fathers in our study. Two fathers had no desire to have children. Although the pregnancies were a surprise, they were not unwanted. Jesse (23, 1 child, co-habitating) was the only father who expressed initial distress and insecurity: “You’re suddenly thrown into a whole new world and you have no idea what’s happening to you”. Eight fathers wanted to have children, though their desires and practices differed. Three fathers had explicit desires to have children. They felt they had their affairs in order and stopped using contraceptives, because they had a stable relationship, were co-habitating and had jobs. Martino (25, 1 child, married) felt overjoyed with his child, because he was trying for a baby: “I wanted to be a dad when I was 18. For me it worked out perfectly. Everything I had imagined came true”. Daan (20, 2 children, co-habitating), however, was still surprised, despite his explicit desire.

I’ve always said I wanted to have kids young. I’ve always felt like I was a bit ahead of my friends, you know. My stepbrother even said, it’s about time now, bro, you cannot stay behind. Our kids have to be able to play together. […] We knew it could happen, but not that it would happen so soon.

Other fathers had latent desires and were not very consistent with contraceptive use. Lorenzo (25, 2 children, co-habitating) said: “It wasn’t completely unexpected, but it wasn’t planned either. We have been together for a longer time and weren’t being careful anymore.” Tim (19, 2 children, co-habitating) had a similar explanation: “for me it was just go with the flow”. These quotes reflect an age norm that youngsters should be settled before they can start a family. These young men who wanted to have children, explained themselves as having their lives in order and being in stable, long-term relationships. Another explanation emerges from comparisons with peers beyond their age group and considering themselves as ahead of their friends. This indicates that these fathers do not perceive youth in age or years, and explain their ‘deviation’ from the age norm in terms of developmental stage and perceived maturity.

Additional age norms can be found in the advantages of young fatherhood. Most fathers highlighted greater physical energy and a longer shared life course with their children, as Jesse (23, 1 child, co-habitating) says: ‘I can still keep up with [my son]”. Another advantage most fathers described was that their fatherhood was the incentive for personal growth and maturity. Ricardo (23, 1 child, single father) shared: “As a person, as a man, as a father and just in my own personality I have grown”. This development was associated with self-regulation, which showed in “giving up crazy stuff” (Jayden, 28, 2 children, co-habitating), “behaving less reckless” (Myron, 23, 1 child, co-habitating), and “party less” (Dylan, 29, 1 child, co-habitating). The age norms that can be discerned here are of youthly energy, physical fitness, risk-taking and partying, while fatherhood is constructed as development into maturity. The energy and fitness of being young match with fatherhood, while the recklessness and craziness do not align with the image of mature fathers.

### Responsibility Through Providership and Care Work: Gender Norms On an Individual Level

The previous section focused on age norms on an individual level, perceived by the young fathers themselves. This section adds to it by highlighting gender norms that appear from the young fathers’ dialogues. A first norm that appears is responsibility. Most fathers (*n* = 8) explained a strong sense of responsibility in traditional practices associated with masculinity. Providing financially was a key part of being a responsible man and father. They believed it was their responsibility that the family had enough money for basic needs, as well as for the future and in case something happened. Others emphasised protection and security as core fathering duties, ensuring that the child has a home to live in and there is enough food. Kevin (22, 2 children, co-habitating), for example, said: “No one should touch my children. No one should touch my family either, because I am the man of the house. […] The woman cleans and does the laundry”. Such fathers emphasised that care and household tasks lie with women, confirming the traditional division of gender between husband and wife, whereby it is a father’s responsibility to provide for the family financially. These perceptions align with traditional masculinity norms, as the fathers explicitly point out their role as a man to keep the family safe and protect them.

However, another gender norm that can be discerned is of care work. Most fathers (*n* = 7) who claimed financial providership and protection as fathering responsibilities, also explained their fathering practices in terms of care work. Child care is one aspect of care work a father should do. Tim (19, 2 children, co-habitating) mentioned: “I know about myself that I am good to the children and that I take good care of them and everything”. Child care included feeding and bathing the children and cleaning up after them. Fathers like Tim want to provide stable, loving environments for their children and ensure that they are happy and have everything they need. Myron (23, 1 child, co-habitating) said: “Yeah, well, that’s what every parent does, right? Just taking care of your child and doing your best with their upbringing to prepare them for the real world.” Fathers perceive childcare also as building resilience of their children, and at the same time letting them be children without worries.

Lorenzo (25, 2 children, co-habitating) is a stay-at-home dad and his partner has paid employment outside of the house. For his family it was “a well-considered choice” that he takes care of the children. Because of his choice not to be the breadwinner, his fathering practices are not based on traditional gender roles. Yet Kevin (22, 2 children, co-habitating), who said that he was the man of the house, described care in terms of the trusting relationship he has built with his child: “I am proud that I have cared for everything, that he was happy as a baby […] and comes to me with trust when something has gone wrong”. Care work is therefore also relational work in developing a good relationship with your child, but also with your partner. Daan (20, 2 children, co-habitating) for example, reflected on himself and his partner as a couple and is happy with “how we are doing things and have managed everything.” Fathers like Daan describe the relationship they have built with their partners and how they have developed their parenthood together also as care.

The care work the fathers mention as their fathering practices are childcare and household activities next to relational care work with their children and partners. Although these activities are forms of care, for these young fathers they do not challenge traditional ideals of masculinity, but co-exist with them. On a symbolic level, the fathers do not perceive care work as less masculine than traditional notions of masculinity such as providership. These fathers’ caring practices are not presented as deviating from dominant notions of what it means to be a man or a father, but are instead framed in ways that remain compatible with conventional masculine norms, such as the responsibility for and protection of the child, and managing things as partners.

### Ambiguous Compliments and Encouragement: Community Perceptions of Young Fatherhood

The previous sections examined age and gender norms as perceived by young fathers on an individual level. This section builds on this by illustrating how young fatherhood is perceived by others in their informal networks and wider community. Many young fathers noticed how strangers question them. Kevin (22, 2 children, co-habitating) said that he and his partner often overheard strangers saying things like ‘look they are young' or ‘why do they have children?’ The fathers were tired of hearing about ‘having a daddy day’, when they are spending time with their children. This implies that it is special for men to care for their children. Myron (23, 1 child, co-habitating) responded to people who asked him about ‘daddy days’ with: “No it’s not a babysitting day, I’m his dad. I’m taking care of my child.” He further explained that “expectations about a father being the breadwinner who needs to work are often still present, especially in the older generations of my parents.” Nevertheless, there are also people who seem to have a more positive attitude towards ‘young’ fathers. Ricardo (23, 1 child, single father) said that some people found it cute when he was grocery shopping or walking with his child: “Some people look at me and at my child and then they nod, like, yes man!” This points out that the traditional gender norms of fathers as providers and mothers as carers are changing. The compliments seems positive, but fathers feel stigmatised, because this suggest that they are doing something special, while they are just like any other parent. Such ‘compliments’ confirm the norm that youngsters are not supposed to be fathers.

Stigma also comes from relatives and friends. Ricardo (23, 1 child, single father) revealed that his father and his best friend repeatedly urged him “to be there for his child”. He knew they meant well, which is why he paid no further attention to it. But the first time they told him ‘to be there’, he thought: “How dare you? What the *?” This illustrates that at the symbolic level, values that are attached to these norms can create stigma. Urging the father to be there can be meant as encouragement, but it also implies that the father is going to run off, which is not on his mind. These examples show that by either questions or compliments from others in their community the fathers are regarded as deviating from what is commonly expected. As *young* fathers, through the intersection of fatherhood with age, they are assumed to be absent and uninvolved. They are either too young to be engaged fathers, or act too responsible as youngsters. Negative fathering behavior is implied because of their youth, pointing towards a supposed lack of responsibility as fathers.

### “You Don’t Have to Hold Everything In”: Changing Norms Within the Community

For three young fathers, their age and gender also intersect with their ethnicity. They are men of colour, born in the Caribbean and moved to the Netherlands in their teenage years. They raised their children in a different cultural context than the one they grew up in, which presents certain challenges. They explicitly described the stigma on their ethnic background in relation to their age and fatherhood. Ricardo (23, 1 child, single father) noticed people staring at him while walking down the street with his child.

There are moments that I walk with him to the store and people are looking like wtf. You know you don’t see a guy of my colour walking behind the stroller, and when you do, 9 out of 10 times the mother is there too. But I walk around with him on my own […] Maybe they think he’s not mine, I don’t know. I’m just walking with my little one, what’s weird about that?

He was angry that people thought the child might not be his, because of his colour and age and even because there was no woman walking next to him. His account reflects how ethnic background, age, and gender intersect in public space, where young fathers of colour are assumed to be absent or irresponsible.

This intersection is also expressed in the taboo about vulnerability for men. These fathers described how masculinity norms in their cultural backgrounds discouraged emotional expression. They said that women were more gentle and sweet, and men were more reserved and did not talk about feelings much. Martino (25, 1 child, married) said: “We men are not allowed to cry and talk about our problems. You take your problems to the grave.” This indicates taboo on showing your emotions to others as a father of colour, which also upholds a masculinity norm of a strong man who controls his emotions. But young fathers are challenging this norm, as Ricardo explained (23, 1 child, single father): “I can cry, and I want to pass that on to my son. You don’t have to hold everything in just because you’re a man." Myron (23, 1 child, co-habitating) explained: “I want it to be different for my child.” These fathers had broken taboos by emphasising the importance of emotional expression in the upbringing of their children. A shift can thus be seen in expressions of masculinity for fathers.

Even though the fathers of colour said that not talking about or showing emotions is linked to their culture or colour, White fathers also referred to a gender norm of the strong man. They also said they had learned not to cry or speak about their emotions, while at the same time most fathers (*n* = 7) described their friends as emotionally supportive. For instance, some said their friends complimented them or expressed pride in how they handled parenting. Two fathers knew they could “always reach out” (Myron, 23, 1 child, co-habitating) and “talk about struggles” with their friends (Martino, 25, 1 child, married). Tim developed a softer side in connecting with his family: “Parenthood has changed my life, in a positive way. I have a completely different goal. I can still be hard, but not for us, not for my family.” This reflects a change in the traditional norms around masculinity; the public image of a man as tough and strong, in contrast to a private side of a soft, caring father.

### Praise for Fathers Versus Focus On Mothers: Professional Attitudes Towards Young Fathers

The previous sections illustrated that in young fathers’ informal network of family and friends and in the broader community, contrasting views on young fatherhood prevail. This section reveals that this also applies in young fathers’ formal professional support networks. Six fathers in this study have received professional support from youth care professionals and all have received perinatal care (with the mother of their children) and have accessed child health care services. They received positive feedback from social work professionals, like telling them that they are taking good care of their children, that they are doing a good job, or expressing specific compliments on the cleanliness of the house or how they have grown as fathers and partners. Dylan (29, 1 child, co-habitating) explicitly stated that others should not be afraid to ask professional support: “You shouldn’t be afraid to get professional help, because otherwise it wouldn’t be called help”. They appreciated having someone who listened en who wanted to take time for them. Kevin (22, 2 children, co-habitating) said: “[Name professional] really helped us, instead of merely going through the motions”. Fathers like him also valued being taken seriously, when they felt at their weakest as a family.

However, some fathers had more negative associations with professional support. They felt not taken seriously as fathers and noticed that all attention was focused on mothers caring for children. Myron (23, 1 child, co-habitating), who received support from youth care professionals later in his life, explained that also earlier: “I needed and would have welcomed support when I had a rough time. The municipality offers a lot of help, mentally and all that, to mums, and mums are welcome when they have problems, but dads don’t have that.” Lorenzo (25, 2 children, co-habitating) said that because of being young, the perception about them as parents was quickly biased: “They assume you can’t do it, you know, because it’s your first child and you’re young parents, but that doesn’t mean that you don’t have experience.” Fathers also said that professionals quickly assumed that the mother would take care of things for the children, because she was thought to be home, while the father also wanted to take time off from work for an appointment. Sometimes professionals even assumed that the mother was a single mother, which was not the case, only because the father could not take time off from work. Martino (25, 1 child, married) noticed that, even when he was present, the professional focused only on the mother and did not look at him or talk to him: “I was just sitting there”. He complained that it is considered special when he goes to the child health clinic while for her it is seen as normal: “No, it’s just a father who takes his child to the children’s health clinic. That is something normal. Isn’t it crazy that people praise that? Like, that’s discrimination […] Because why is all that so praised for a man?”

These findings indicate experiences of negative and positive gendered stigma. Fathers are treated positively or are ignored by professionals. Although being treated well is appreciated, being treated overly positively evokes suspicion and irritation among young fathers. This reflects the unequal valuation of gender norms, in which fathering is treated as special and mothering as self-evident. Yet, it also shows that gender norms are changing. Despite growing awareness that men (can) care for children, this recognition is not structurally supported by professionals nor reflected at institutional and policy levels. Positive intentions of professionals may create ambivalence or reinforce feelings of vulnerability, rather than diminishing them, as the following paragraph illustrates.

Young fathers experience anxiety and fear of doing something wrong as a parent (*n* = 6). They explicitly mentioned the fear of failure, which especially exists among among *young* parents. Kevin (22, 2 children, co-habitating) said: “I was afraid I couldn’t do it, that something would happen.” These young fathers experienced much stress and anxiety living with the thought that “even with the slightest mistake, the authorities would take my child away from me. A lot of men around me share this fear. There are a lot of horror stories” (Myron, 23, 1 child, co-habitating). Dylan (29, 1 child, co-habitating) shared that he always tried to manage it all by himself, pretenting he did not need any help. He was afraid that if he showed weakness, professionals might think he could not do it and he would lose his child. Myron, Kevin and Dylan were in a relationship with the mothers of their children, but single father Ricardo was co-parenting and feared his child might be taken away by child protection services if he made a mistake. He was even hesitant to make plans on days when his child was with his ex-partner, to ensure he remained available and was not perceived as a bad father should she call on him for childcare if needed. Traditional gender norms expressing that men should not fail nor be vulnerable play a role here, alongside the motherhood ideal that cultivates images where children are legally assigned to the mother as main carer when parental relationships fail. For young fathers, there is even more at stake, since their age makes them subject to more stigma. On personal and institutional level, intersecting gender and age norms contribute to young fathers’ fear of being judged as “bad” fathers.

## Conclusion and Discussion

This study has examined how young fathers in the south of the Netherlands experience their fatherhood in relation to age and gender norms. Young fathers’ narratives have revealed an interplay of age, gender and ethnicity norms. The age norms at play are those of youngsters as energetic, physically fit, risk-taking and partying. Young fathers deviate from these norms as they perceive themselves ahead of their peers, having settled down and being in a stable relationship, which accounts for their desire for children and having children at a young age. Not their age, but their development and maturity allow them to be a father. Even though they can tap into their youthly conduct of energy and fitness, having put behind them the crazy party mode makes them mature fathers. This contrasts what Beggs Weber (2020) found in her study on young fathers who still go out and get a little wild. Nevertheless, the young fathers in her study also negotiate age and gender discourses to show that they have stepped up and become a man (Beggs Weber, 2020). This is in line with the social engagement frame rather than the social problems framework of young fatherhood (Neale & Tarrant, 2024). It shows that by deviating from as well as adhering to youth norms, the young fathers perceive themselves as responsible fathers.

Care work that the young fathers identify are child care practices and relational care work with their children and partners to develop a strong relationship. This ties in with studies on engaged fatherhood (Adler, 2023; Eerola et al., 2025; Hodkinson & Brooks, 2023). On the spectrum from caring, involved and engaged fathers to authoritarian, emotionally distant and absent fathers, the young fathers cannot be positioned on one or the other end. They present their caring practices as part of their responsibility as a father and man to tend to the needs of their family, and as such align them with norms of providership. Care work thus is not valued as less than providing financially, but is included in a notion of responsible fatherhood. This confirms other research saying that care practices include direct childcare and indirect care by being financially responsible (Doucet, 2025). Paid and unpaid work are increasingly seen as interconnected rather than oppositional, with paid work supporting unpaid care and thus functioning as a form of indirect care (Hanlon, 2024; Doucet, 2025). Nonetheless, critics argue that this does not constitute an equal division of care, as domestic and childcare labour remain unpaid, and fathers may be doing more care work without necessarily assuming an equal share of the mental load (Wojnicka & De Boise, 2025). As this was not focused on in our study, more research on whether this applies for *young* fathers would be relevant.

As such, this study adds to international scholarship on a change in traditional masculinity norms (Eerola et al., 2025; Elliott, 2016; Grau Grau et al., 2022; Roberts & Prattes, 2024). Some fathers are actively changing masculinity norms, by rejecting the taboo on expressing emotions. They want to teach their children that men should show their emotions and they welcome emotional support from friends. This reflects a shift in traditional views on masculinity, in which the public ideal of the tough, strong man increasingly coexists with a more private identity centred on care and emotional involvement in fatherhood (Hunter et al., 2017). Young fathers experience a normative change through positive remarks they receive from others within their community. Whereas mothers’ caring activities are taken for granted, young fathers’ caring activities are overly complimented. The fathers feel like they are doing what any parent should do. Although the community and division of tasks, as described by Van der Pas (2016), hold potential for parenting support, they also reflect unequally valued norms that can undermine parenting support. Compliments point to young fathers’ deviancy from the norm and evoke stigma. These experiences are particularly noticeable for fathers of colour, who notice that when their age and ethnicity intersect with their identity as men, they are often assumed to be absent and uninvolved fathers, despite the caring activities they perform.

Among the young fathers of this PAR project, one was a stay-at-home dad. Although caring fatherhood is becoming more normalised, fathers as primary caregivers, whose paid work is not central, are still an exception (Gallais, 2023). Care activities are not equally distributed between parents, as gendered care practices persist, shaped in part by institutional and organisational policies (Adler, 2023; Hodkinson & Brooks, 2023; Martínez-Pastor et al., 2024). The ambivalent views on young fatherhood that the fathers experience on an individual level within their community, also prevail on an institutional level with support from professionals. Young fathers have received positive feedback on their childrearing practices and meaningful support on their relationship from professionals. However, in relation to gender, professionals often treat young fatherhood as exceptional or overlook young fathers even though they are present, while motherhood remains taken for granted. In relation to age, professionals assume that young fathers cannot do or do not know something. Like other research (Davies, 2016; McGirr et al., 2020; Mniszak et al., 2020; Webb et al., 2023), this study shows that unintended barriers in services make engagement harder, especially for young fathers who feel out of place.

Young fathers’ fear of being judged as ‘bad’ fathers, because of their age intersecting with their fatherhood, reinforces vulnerability rather than reducing it. The inherent vulnerability of every parent that Van der Pas (2016) assumes, overshadows the ultimate responsibility of parents that is also assumed. Fathers have more at stake than mothers, because of the mother-centred culture that prevails in social work policy and practice. Even though the fathers in our study perceive themselves as responsible, caring fathers, in general fathers are rarely seen as capable caregivers (Gallais, 2023). As Gallais (2023) states, unequal relationships between men and women in childcare often lead to fathers being treated as the second parent who knows less. Relating this to the (usually unintentional) thresholds of social work services in reaching and engaging with young fathers (Baum, 2017; Davies, 2016; Mniszak et al., 2020), the much desired connection between young fathers and social work professionals is not self-evident. This gap is not only a matter of insufficient outreach or engagement, but is embedded in institutional norms that continue to centre motherhood and treat fatherhood as optional or peripheral.

Even though this study has taken an intersectional approach on gender, age and ethnicity, this could have been taken a step further by also exploring the economic position or class of the fathers. Fathers with independency in work who can make their own choices and fathers with partners who work full-time, can easier make work adaptations to embrace child care (Gallais, 2023; Martínez-Pastor et al., 2024). Men who meet the norms of the masculinity ideal are often the ones who can afford to be involved in care work (Gallais, 2023; Hunter et al., 2017). Young fathers like the ones in our PAR project, on the other hand, are generally not in a socioeconomic position to make such choices. This impacts on how they can practice their care work and how they reflect on their fatherhood. It builds on other studies stating that gender sensitivity matters greatly when working with fathers (Baum, 2017; Pfitzner et al., 2020), by adding the need for an age-sensitive approach in social work and in particular youth care (Ponzoni et al., 2021).

As Tarrant & Neale (2024) point out, most studies on young fatherhood have limited samples, just like ours. It is therefore important to be cautious about generalising research findings to other populations of young fathers. Van de Beek et al. (2025a) conducted research that generated insight on young fatherhood in a Dutch health care setting, particularly perinatal care. Our study in social work complements theirs by demonstrating that young fathers feel unrecognised by professionals. Further similarities are that professional practice, systems and policies are ill-equipped to support fathers in general, but specifically young fathers, since professionals (unintentionally) reproduce traditional norms on gender and age (Van de Beek et al. 2025a, b). Our study thus contributes to a growing body of knowledge in the Netherlands on (young) fatherhood that connects the fields of health care and social work.

Gender was studied as a theme, but also played a role in conducting the research. The unequal gender balance in the PAR team of researchers and professionals reflected the gender balance in the women dominated field of social work. All dialogue sessions were also conducted by women. This may have influenced the responses of the respondents, since obstacles of fatherly involvement in social work services are the large amount of women that fathers interact with and the communication differences between them (Baum, 2017). This was mitigated by setting up the dialogue sessions as reciprocal and accessible, as discussed beforehand by the advisory group including a young father and mother. In the dialogue sessions, the fathers noticeably expressed their emotions and shared experiences of stigma, taboos and their desires to have children. Nevertheless, the social work field in the Netherlands, as well as in other countries, could benefit from a more diverse population of professionals. Training programmes to develop gender and age-sensitive attitudes and practices could also be beneficial. Our PAR project has contributed to this. The participatory aspects of the project generated practice-based insights that informed the further development of the support method in youth care, contributing to improved professional engagement with young fathers. These processes supported social workers’ professional learning and fostered more gender-sensitive and father-inclusive practices.

This PAR project has led to actual changes in professional Dutch social work practices, particularly in youth care, through the improved father-inclusive support method and related training programme with a work book for young fathers and mothers and a handbook for professionals. It led to a call for action for more father-inclusive practices in the fields of health care and social work (Petit-Steeghs et al. 2024), and to a joint project in health care and social work with young fathers and professionals in various Dutch municipalities. However, further action is required to advance father-inclusive practices within the field of social work. Engaged and caring fatherhood should be included and normalised in social work services. Social work professionals should be aware of the gender norms they express and acknowledge intersections of gender, age and ethnic background, which Ponzoni et al. (2021) also advocate. Embracing an intersectional approach on gender, age, class and ethnicity can help professionals better align their support with the realities of young fathers, rather than relying on stereotypes on the intersections of masculinity and youth such as the distant provider or incapable young parent. This begins with offering (online) information materials that are accessible and relevant to young fathers, and verbally and visually aimed at them, including images with a variety of fathers in non-stereotypical ways (Borman et al., 2024). Moreover, it is important to always explicitly address fathers and mention fathers when talking about parenthood and care work to other parents, professionals and policy makers.

Our findings point out that engaging young fathers meaningfully in social work services requires more than offering access. It starts with engaging with fathers from the beginning. Professionals should make sure the appointments take place at a time and place that suits both fathers and mothers and have chairs for father and mother that position them as equal partners (Borman et al., 2024). Professionals also need to take time to get to know fathers and mothers equally well, and give equal attention to fathers and mothers in all communication (Borman et al., 2024). Furthermore, professionals could encourage fathers to ask questions, share views and discuss their tasks and activities (Borman et al, 2024). This requires a shift in institutional culture, from viewing fathers as secondary or exceptional to acknowledging them as responsible, caring and emotionally invested parents.

## Data Availability

No datasets were generated or analysed during the current study.
